# Rapid, noncontact, sensitive, and semiquantitative characterization of buffered hydrogen-fluoride-treated silicon wafer surfaces by terahertz emission spectroscopy

**DOI:** 10.1038/s41377-022-01033-x

**Published:** 2022-11-25

**Authors:** Dongxun Yang, Abdul Mannan, Fumikazu Murakami, Masayoshi Tonouchi

**Affiliations:** Institute of Laser Engineering, Osaka University 2-6 Yamadaoka, Suita, Osaka, 565-0871 Japan

**Keywords:** Imaging and sensing, Optical spectroscopy

## Abstract

Advances in modern semiconductor integrated circuits have always demanded faster and more sensitive analytical methods on a large-scale wafer. The surface of wafers is fundamentally essential to start building circuits, and quantitative measures of the surface potential, defects, contamination, passivation quality, and uniformity are subject to inspection. The present study provides a new approach to access those by means of terahertz (THz) emission spectroscopy. Upon femtosecond laser illumination, THz radiation, which is sensitive to the surface electric fields of the wafer, is generated. Here, we systematically research the THz emission properties of silicon surfaces under different surface conditions, such as the initial surface with a native oxide layer, a fluorine-terminated surface, and a hydrogen-terminated surface. Meanwhile, a strong doping concentration dependence of the THz emission amplitude from the silicon surface has been revealed in different surface conditions, which implies a semiquantitative connection between the THz emission and the surface band bending with the surface dipoles. Laser-induced THz emission spectroscopy is a promising method for evaluating local surface properties on a wafer scale.

## Abstract (web summary)

In this paper, the THz emission properties from Si wafers with different surface conditions and doping conditions have been concluded, which show the potential application of LTEM on surface estimation

## Graphical abstract

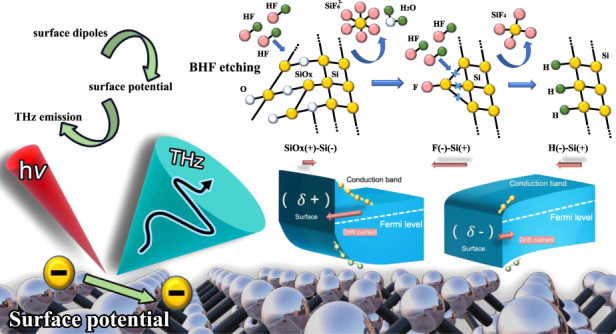

## Introduction

As the most essential semiconductor in the modern electronic industry, silicon (Si) has been the focus for nearly half a century^1^. However, its surface properties remain a mystery that needs to be resolved. Because of the complicated situations at the Si surface, such as the native oxide layer, dangling bonds, or the presence of other absorbed particles, the properties of the Si surface, including the surface potential and surface electric field, vary widely but are essential to Si-based devices fabrication^2,3^. In the modern semiconductor industry, hydrogen fluoride (HF) and its buffered (BHF) solutions are frequently used to treat the surface and remove the native oxide layer^4–6^. After BHF etching, a hydrogen(H)-terminated surface forms, and the surface properties change significantly owing to the variation in the surface states and the generation of surface dipoles^7,8^. Currently, a variety of methods are used to characterize the defects at the wafer surface to confirm a better surface for the next photolithography process in the semiconductor industry^9^. The standard wafer inspection techniques include the brightfield and darkfield inspection by using a laser beam and its reflection at a specific angle^10^, the electron-beam inspection, and multi-beam inspection by using an electron beam for higher resolution^11^. Besides the defects at the surface of Si wafer, the surface electric properties are also important to further fabrication and influence the device quality. To improve the yield of products integrated on the Si wafer, it is important and necessary to characterize the surface properties of the Si wafer rapidly, efficiently, and quantitatively before and after the chemical treatment during the fabrication process. Several useful but complicated tools have been proposed to estimate the surface potential; these include X-ray photoelectron spectroscopy^12^, surface photovoltage measurement^13,14^, and Kelvin force microscopy^15,16^. Methods for sensitive local surface evaluation and rapid surface property mapping are still lacking and urgently needed. Here, we propose laser-induced terahertz (THz) emission spectroscopy (TES) and laser-induced terahertz emission microscopy (LTEM) as the most promising candidate. These are performed as a sensitive and semiquantitative noncontact local characterization method with an additional mapping function^17^ that can efficiently evaluate surface properties, such as surface potential^18^, passivation layer^19^, and surface charge density.

**Fig. 1:**
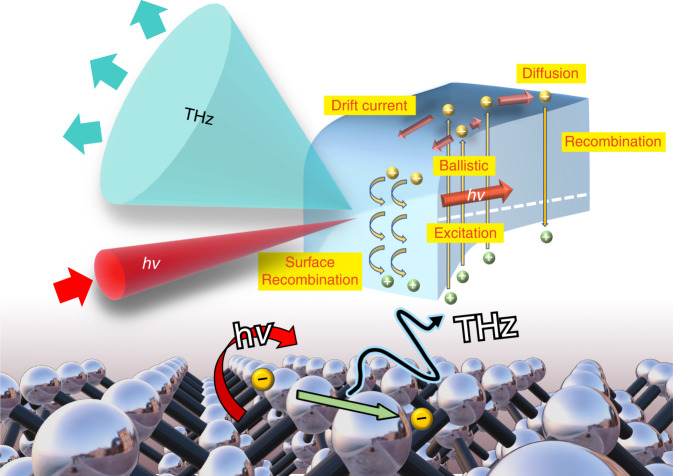
Illustration of THz emission from a semiconductor surface with laser excitation. The THz emission from the semiconductor is due to the ultrafast charge transport including the drift current, photocarrier diffusion and the ballistic.

Ultrafast laser excitation at the surface of a semiconductor generates THz radiation as a result of ultrafast charge transport^20,21^, the mechanism of which can be classified mainly into two categories: 1. photocarrier diffusion including ballistic transport^22,23^, owing to the photo-Dember effect, where the difference in the mobilities between holes and electrons induces a transient photocurrent, which is dominant in narrow-bandgap semiconductors such as InAs^24^, InGaAs^25^, and InSb^26^, and 2. the drift of photocarriers with the surface electric field resulting from the pinning of the Fermi level to the surface, the polarity of which can be flipped by changing the doping type of semiconductor (e.g., GaAs^27^ or InP^28^). A schematic illustration of the THz emission mechanism from the semiconductor surface is shown in Fig. 1. The THz emission from the Si metal–oxide–semiconductor structure is considered to be the combined result of the drift current and diffusion in previous reports^29,30^. On the bare Si surface, the photo-Dember effect is quite weak compared to the drift current resulting from the surface electric field. Therefore, for simplicity, we regard the drift current within the surface band bending area as the main source of THz emission in our discussion.

In the present work, we observe the THz emission from the Si surface before and after the removal of the native oxide layer using a BHF solution and explain the mechanism of the THz emission variation resulting from the change in surface conditions. Meanwhile, the parameters of the doping types and doping concentration also indicate their impact on the observed THz emission waveforms in terms of both amplitude and polarity. The flipping of the THz waveform reveals the strong dependence of the THz emission on the surface band bending, which is dominated by the surface state energy level and Fermi level in bulk. Furthermore, we discuss the parameters of the surface properties and provide an LTEM image of the surface potential distribution on the Si surface with a line-space pattern after BHF etching as an example of the application. LTEM–TES is a promising tool for achieving rapid noncontact and sensitive characterization of Si surface properties and will benefit the modern Si industry.

## Results

**Fig. 2:**
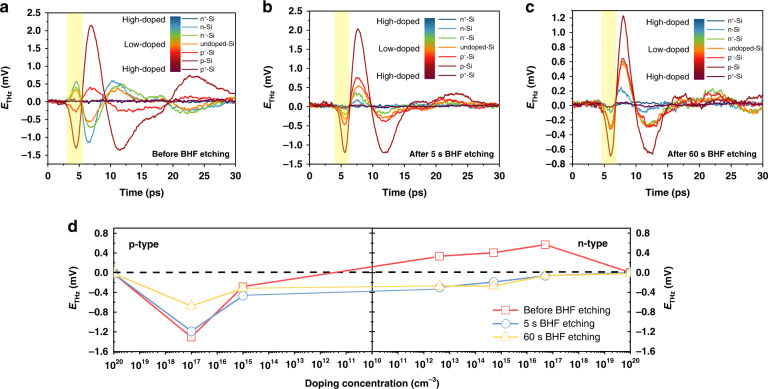
The overall results of the THz emission from Si wafers in different conditions. THz emission spectra from Si wafers of different doping types and doping concentrations: (**a**) before BHF etching; (**b**) after 5 s BHF etching; (**c**) after 60 s BHF etching. **d** Doping concentration dependence on THz emission amplitude (where the first peak is marked in yellow) ranges from high-doped p-type to high-doped n-type Si

First, we measured the THz emission spectrum from the Si samples before and after BHF etching with etching times of 5 and 60 s, as shown in Fig. 2a−c. Due to the large beam diameter with 30° incident angle in our experiment, a long-time duration THz pulse was observed due to the surface charge oscillation and phased-array effect^31^. In order to directly reflect the dynamic properties of the photocarriers at the surface, we focus on the electric field at maximum intensity in the first peaks of the THz emission spectrum^18^, as shown in the yellow area. Before BHF etching, the first peaks of the THz emission spectrum of n-type, n^−^-type, and undoped Si all exhibit a positive polarity, whereas under p- and p^−^-type conditions, the polarity is negative. After BHF etching, the polarity of the THz emission waveform in n-type, n^−^-type, and undoped Si reverses to become negative, while the polarity under p- and p^−^-type conditions remains the same. Under extremely high-doped conditions, the THz emission amplitudes from both n^+^- and p^+^-type Si surfaces are extremely weak before and after BHF etching, which is barely observed in our system. The high doping concentration significantly alters the band structure and decreases both minority carrier lifetime and diffusion length, leading to the weak excitation of the THz emission^32,33^. The thickness of surface potential barrier in the high doping condition is quite short (2~3 nm),which makes the barrier work as a tunneling barrier and become transparent for the carriers to form ohmic contacts^34^, leading to the small drift current and weak THz emission. Meanwhile, the difference in the mobilities between electrons and holes in the high-doped Si is not much, leading to the weak THz emission due to the small photo-Dember effect^35^.

Figure 2d provides the curve of doping concentration dependence on the THz emission amplitude under different surface conditions. Before BHF etching, the amplitude of the THz emission exhibits an approximately linear relationship with the order of the doping concentration. The amplitude decreases with the doping concentration declines and the polarity of the peak reverses under different doping types. After 5 s BHF etching, the native oxide layer is removed, and the surface condition has changed. Correspondingly, the THz emission changes in both amplitude and polarity under n-type, n^−^-type, and undoped Si conditions whereas, under p- and p^−^-type conditions, the amplitude also changes. After 60 s BHF etching, the THz amplitude slightly decreases, especially in the p-type condition. In other words, the relationship curve between doping concentration and THz emission amplitude will shift upward or downward depending on the surface conditions. Meanwhile, we explored the power dependence on the THz emission amplitude of these samples before and after 60 s BHF etching, as shown in Fig. S3a, b. The results of power dependence on THz emission support the fact that the variation of the surface electric field due to the doping conditions and surface dipoles leads to the variation of THz emission in both amplitude and polarity among each laser power condition. The THz amplitude of each sample shows linear relationship with the laser power. The experiment results of the THz emission spectra of samples before and after 60 s BHF etching under different powers are shown in Figs. S1 and S2 in the supplementary information.

**Fig. 3:**
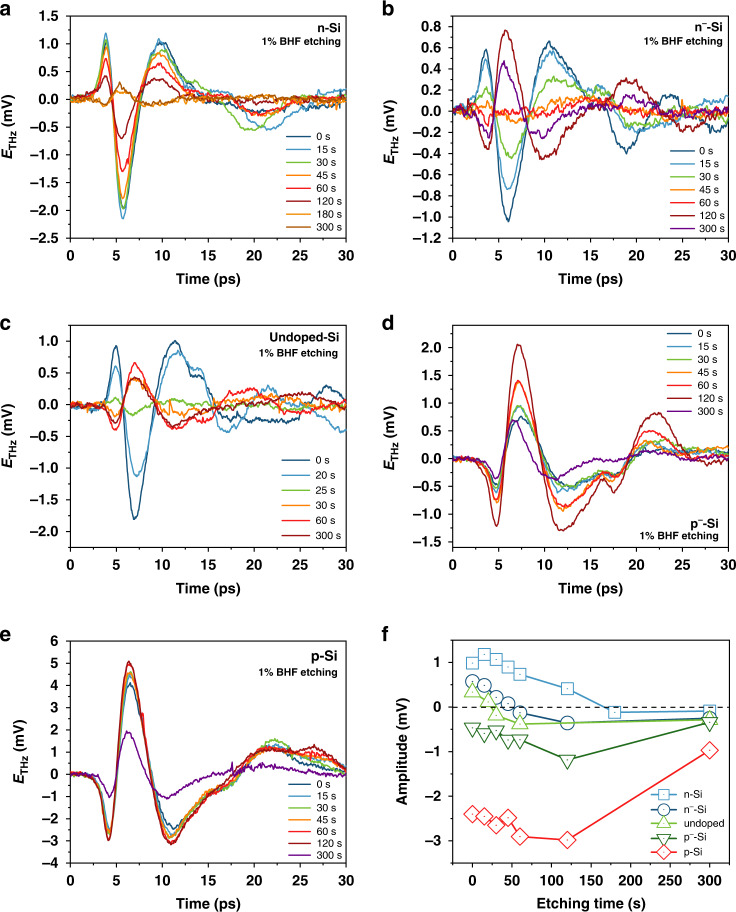
THz emission variation with different BHF etching times. THz emission spectra from Si wafers with different etching times by using a 1% dilute BHF solution: (**a**) n-type Si, (**b**) n^−^-type Si, (**c**) undoped Si, (**d**) p^−^-type Si, and (**e**) p-type Si. **f** Variation of THz emission amplitude (the first peak) with the increase of etching time

We further explored the influence of the surface conditions on the THz emission spectrum from the etched Si samples using a 1% dilute BHF solution under a short etching time interval to observe the rapid variation of the surface condition and illustrate the impact on THz emission. Since the morphology of the Si surface does not largely change after BHF etching, we expect the influence of the slight morphology variation on THz emission can be ignored^36^. Figure 3a−e shows the THz emission spectra for different doping types and doping concentrations after 1% dilute BHF etching, from which we can observe the variation in the THz emission with the change in the surface condition under different doping conditions. For n-type Si, the THz emission from the surface gradually decreases with increasing etching time, and, finally, the polarity of the first peak reverses from positive to negative. A similar variation was observed under n^−^-type and undoped conditions; however, after the reversal of the polarity, the amplitude increased with further BHF etching. For p-type Si, the amplitude first increases with a short etching time; however, after a long etching time, the amplitude decreases, which corresponds to the surface condition variation in which the native oxide layer is removed and the surface becomes fluorine(F)-terminated as the mid-gap stage and finally becomes H-terminated^37^. The variation in THz emission amplitude of the first peak is shown in Fig. 3f. We also measured the THz emission spectrum from the H-terminated surface of n^−^-type Si with long-time laser illumination, as shown in Fig. S4 in the supplementary information. The results indicate that the H-terminated surface is quite stable under laser illumination and the decline tendency of the amplitude with illumination time increases is due to the long-time dissociation of the hydrogen from the surface^38^.

THz emission is extremely sensitive to surface band bending. When the ultrafast laser pulse illuminates the Si surface, the photocarriers will be excited owing to the extra energy between the photon energy and bandgap. When band bending occurs, the photocarriers are accelerated by the surface electric field and move apart to generate an ultrafast photocurrent, which excites the THz emission from the surface. When the penetration depth (*λ*) is greater than the thickness of the built-in field, the THz emission field $E_{{{{\mathrm{THz}}}}}$ can be simplified as^30^.

1 $$\begin{array}{*{20}{c}} {E_{{{{\mathrm{THz}}}}} \propto \mu \frac{{V_{{{\mathrm{s}}}}}}{\lambda }I_{{{\mathrm{p}}}}} \end{array}$$

where *μ* is the carrier mobility, $V_{{{\mathrm{s}}}}$ is the surface potential, and $I_{{{\mathrm{p}}}}$ is the laser power intensity. This relationship indicates that the polarity of the surface band bending determines the polarity of the waveform in the THz emission spectrum. In the next section, we discuss the influence of different surface conditions on the THz emission and semi-quantitatively estimate the parameters related to the surface dipoles, surface potential, surface state energy level, and surface charges.

## Discussion

### Surface dipoles

The surface dipoles play an important role in THz emission from the Si surface^8,39^. These create a field that opposes further electron transfer into the vacuum and changes the surface energy between the Fermi level and the vacuum level^40^. After BHF etching, the native oxide layer is removed from the Si surface, and an H-terminated surface finally forms, which leads to the surface dipoles. The process of BHF etching and its influence on the surface properties are discussed here.

The BHF solution is a mixture of HF and ammonium fluoride (NH_4_F) (BHF = HF + H_2_O + NH_4_F), which provides sufficient F^−^ ions and prevents the depletion of fluoride ions. During the etching process, the following chemical reaction occurs:

$${{{\mathrm{SiO}}}}_2 + 6{{{\mathrm{HF}}}} \to 2{{{\mathrm{H}}}}^ + + {{{\mathrm{SiF}}}}_6^{2 - } + 2{{{\mathrm{H}}}}_2{{{\mathrm{O}}}}$$

**Fig. 4:**
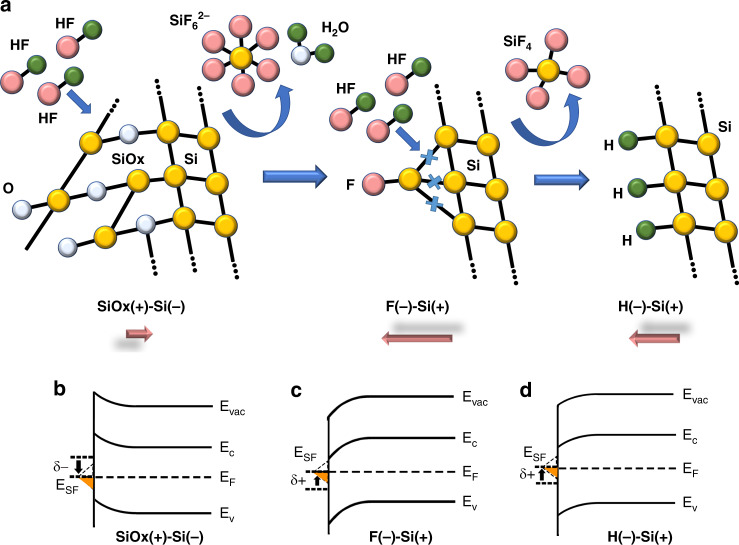
The BHF etching process and band diagram variation. **a** Chemical process on the Si surface during BHF etching and the surface dipole moment resulting from different electronegativities of surface atoms. The native oxide layer is removed, F-terminated Si is generated as a mid-stage, and the H-terminated surface is finally formed as the stable condition. The effect of the surface dipoles on the band diagrams of the (**b**) n^−^-type Si surface with a native oxide layer, (**c**) F-terminated surface, and (**d**) H-terminated surface

With BHF etching on the Si surface, the Si–O bonds are replaced by Si–F bonds and form the F-terminated surface at first as a mid-stage. Subsequently, the Si–Si bonds are inserted by further HF attacks to generate SiF_4_ away from the surface, leaving behind an H-terminated Si surface^37^. This process is illustrated schematically in Fig. 4a. Many studies on the Si surface with HF or BHF etching have been reported^37,41,42^. G. W. Trucks et al.^37^ from Bell laboratory reported the mechanism of the HF etching on Si surface and the existence of F-terminated and H-terminated conditions based on the first-principles solid-state calculations and Paul.G Spizzirri from Monash university proved the existence of Si-H and H-Si-F at the Si surface after HF etching by using probe enhanced, nano-Raman spectroscopy (PERS)^43^. In our work, the variation in the surface condition is reflected by the THz emission spectrum with different etching times. Because of the different electronegativities of these atoms, listed in Table 1, the distribution of the electrons at the surface forms surface dipoles. These surface dipoles apply an additional potential and move all the levels of a species (e.g., the surface state energy level) located outside the dipole layer relative to the vacuum energy level. Therefore, a positive potential (δ) moves the surface state energy levels toward the vacuum energy level, whereas a negative δ moves the outside levels away from the vacuum energy level, leading to a variation in the Fermi levels at the surface^8^. Here we take n^–^-type Si as an example to explain the impact of the surface dipoles.

**Table 1:** Electronegativity of elements and compounds

	H	F	SiO_x_	Si
Electronegativity	2.8	4.0	1.7	1.8

In the initial condition of the n^−^-type Si surface, the native oxide layer at the surface includes positive fixed charges and forces electrons away from the surface. This induces a negative δ and moves the surface Fermi level downward, as shown in Fig. 4b. As a result, upward surface band bending occurs because of the Fermi level pinning at the surface. After the removal of the native oxide layer, the surface is terminated by the fluorine atoms in a short time of BHF etching, and a positive δ is induced at the surface owing to the different electronegativities between the fluorine and Si atoms. With a surface dipole layer, the surface Fermi level approaches the conduction band and results in downward band bending at the surface, as shown in Fig. 4c. After a long etching time, the F–Si bonds get replaced by H–Si bonds at the surface as a stable condition. The electronegativity of hydrogen is greater than that of Si but lower than that of fluoride, leading to a lower surface band bending in the H-terminated surface condition, as shown in Fig. 4d. The experimental results strongly support the deep connection between the variation of band bending resulting from BHF etching and the variation of the THz emission in both polarity and amplitude. According to the experimental results in Figs. 2, 3, the polarity of the THz emission indicates the direction of the surface band bending, and the amplitude reflects its intensity.

### Doping types and doping concentration

**Fig. 5:**
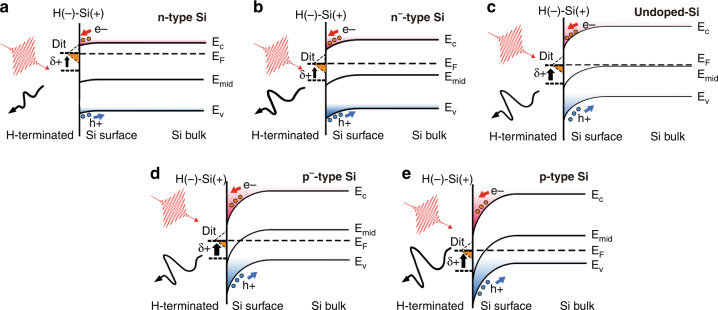
Band diagrams of H-terminated Si surface. Band diagrams of the H-terminated Si surface with different doping types and concentrations: (**a**) n-type Si, (**b**) n^−^-type Si, (**c**) undoped Si, (**d**) p^−^-type Si, and (**e**) p-type Si. Because of the surface dipoles from the H–Si bond at the surface, the surface state energy level changes with +δ and leads to surface band bending

In the bulk region of Si, the Fermi level is determined by the doping type and concentration. On the surface, the periodicity of the crystals is disturbed. Dangling bonds, defects, and impurities cause surface states with energy levels within the bandgap, and surface dipoles also influence the surface Fermi level. Therefore, the Fermi energy-level difference between the surface and bulk causes surface band bending^44^. Watanabe et al.^13^ reported that, after HF etching, large band bending was observed at the p-type Si surface, whereas an almost flat band was observed in the n-type condition, using photoelectron spectroscopy. The donor surface states at the H-terminated Si surface are responsible for the band bending. Schlaf et al.^45^ also reported Fermi pinning on a BHF-treated Si surface. Figure 5 shows the band diagrams of the H-terminated Si surface with different doping types and concentrations; these are consistent with previous research results^13,45^. After BHF etching, the Fermi level at the surface is pinned close to the conduction band owing to the surface dipoles. In the n-type Si condition (Fig. 5a), the Fermi level in bulk is located close to the conduction band, and the energy-level difference between the bulk and surface is relatively small; therefore, band bending is barely observed. In the p-type condition (Fig. 5e), the Fermi level in bulk is close to the valence band and far from the conduction band, leading to a large band bending at the surface. Therefore, when the Fermi level in bulk moves with the doping type and concentration, a variation in band bending occurs as well; this is reflected by the variation of the THz emission amplitude from the Si surface. The band diagrams of the Si surface with the native oxide layer and the F-terminated surface are provided in the supplementary file, as shown in Figs. S5, S6.

### Surface charges (*Q*_ss_ and *Q*_sc_) and surface potential (*V*_s_)

The surface charges in the surface states cause a nonequilibrium carrier density at the surface, leading to a surface electric field and potential. The carrier density in the vicinity of the surface deviates from its equilibrium value and results in the surface space charge region (SCR), which obeys the charge conservation rule^44^:

2 $$\begin{array}{*{20}{c}} {Q_{{{{\mathrm{ss}}}}} = - Q_{{{{\mathrm{sc}}}}}} \end{array}$$

where *Q*_ss_ is the net surface charge and *Q*_sc_ is the net charge in the SCR. Based on Poisson’s equation, the relationship between the surface potential (*V*_s_) and the surface electric field intensity (*E*_s_) can be easily obtained as

3 $$\begin{array}{*{20}{c}} {E_{{{\mathrm{s}}}} = \pm \frac{{\sqrt 2 kT}}{{qL_{{{\mathrm{D}}}}}}F\left( {\frac{{qV_{{{\mathrm{s}}}}}}{{kT}},\frac{{n_{{{{\mathrm{p}}}}0}}}{{p_{{{{\mathrm{p}}}}0}}}} \right)} \end{array}$$

where $L_{{{\mathrm{D}}}} = \left( {\frac{{\varepsilon kT}}{{q^2p_{{{{\mathrm{p}}}}0}}}} \right)^{1/2}$ is the extrinsic Debye length, $\varepsilon = \varepsilon _0\varepsilon _{{{\mathrm{r}}}}$ is the dielectric constant of the semiconductor, $n_{{{{\mathrm{p}}}}0}$ and $p_{{{{\mathrm{p}}}}0}$ are the carrier densities of electrons and holes, respectively, *q* is the elementary charge, *k* is the Boltzmann constant, and *T* is room temperature. The polarity of *E*_s_ indicates the electric field direction. The term $F\left( {\frac{{qV_{{{\mathrm{s}}}}}}{{kT}},\frac{{n_{{{{\mathrm{p}}}}0}}}{{p_{{{{\mathrm{p}}}}0}}}} \right)$ can be expressed as follows:

4 $$\begin{array}{l} F\left( {\frac{{qV_{{{\mathrm{s}}}}}}{{kT}},\frac{{n_{{{{\mathrm{p}}}}0}}}{{p_{{{{\mathrm{p}}}}0}}}} \right) = \left\{ \left[ \exp \left( { - \frac{{qV_{{{\mathrm{s}}}}}}{{kT}}} \right) + \frac{{qV_{{{\mathrm{s}}}}}}{{kT}} - 1 \right]\right. \\ \left.\qquad\qquad\,\,\,\,\qquad+\, \frac{{n_{{{{\mathrm{p}}}}0}}}{{p_{{{{\mathrm{p}}}}0}}}\left[ {\exp \left( {\frac{{qV_{{{\mathrm{s}}}}}}{{kT}}} \right) - \frac{{qV_{{{\mathrm{s}}}}}}{{kT}} - 1} \right] \right\}^{\frac{1}{2}} \end{array}$$

According to Gauss’s law, the charge density in the SCR ($Q_{{{{\mathrm{sc}}}}}$) is calculated from the electric intensity at the surface ($E_{{{\mathrm{s}}}}$) as^46^

5 $$\begin{array}{*{20}{c}} {Q_{{{{\mathrm{sc}}}}} = - \varepsilon E_{{{\mathrm{s}}}} = \mp \frac{{\sqrt 2 \varepsilon kT}}{{qL_{{{\mathrm{D}}}}}}F\left( {\frac{{qV_{{{\mathrm{s}}}}}}{{kT}},\frac{{n_{{{{\mathrm{p}}}}0}}}{{p_{{{{\mathrm{p}}}}0}}}} \right)} \end{array}$$

The surface state density (*N*_t_) is composed of the donor and acceptor states within the gap, which can be approximately calculated as $D_{{{\mathrm{v}}}}(E) = N_{{{\mathrm{t}}}}e^{ - (E - E_{{{\mathrm{v}}}})/b^2}$ and $D_{{{\mathrm{c}}}}(E) = N_{{{\mathrm{t}}}}e^{ - (E - E_{{{\mathrm{c}}}})/b^2}$. The total surface charge density can then be derived from the surface states as a function of the bulk Fermi-level position throughout the bandgap^47^:

6 $$\begin{array}{*{20}{c}} {Q_{{{{\mathrm{ss}}}}} = e\mathop {\int}\limits_{E_{{{\mathrm{v}}}}}^{E_{{{\mathrm{c}}}}} {\left( {1 - F\left( {E_{{{\mathrm{t}}}}} \right)} \right)D_{{{\mathrm{v}}}}(E){{{\mathrm{d}}}}E - e} \mathop {\int}\limits_{E_{{{\mathrm{v}}}}}^{E_{{{\mathrm{c}}}}} {F\left( {E_{{{\mathrm{t}}}}} \right)D_{{{\mathrm{c}}}}(E){{{\mathrm{d}}}}E} } \end{array}$$

where $E_{{{\mathrm{c}}}},$ $E_{{{\mathrm{v}}}}$, and $E_{{{\mathrm{t}}}}$ are the conduction band, valence band, and surface state energy levels relative to the mid-gap energy level, respectively. Table 2 provides the surface state energy level for different surface conditions and the parameters for the surface state density^48^.

**Table 2:** Parameters used in the calculation^8,49^

Parameter	*N*_t_ (cm^−2^ eV^−1^)	*b* (eV)	*E*_t(Si–SiOx)_(eV)	*E*_t(Si–F)_ (eV)	*E*_t(Si–H)_ (eV)
Value	4 × 10^12^	0.2	−0.1	0.45	0.42

**Fig. 6:**
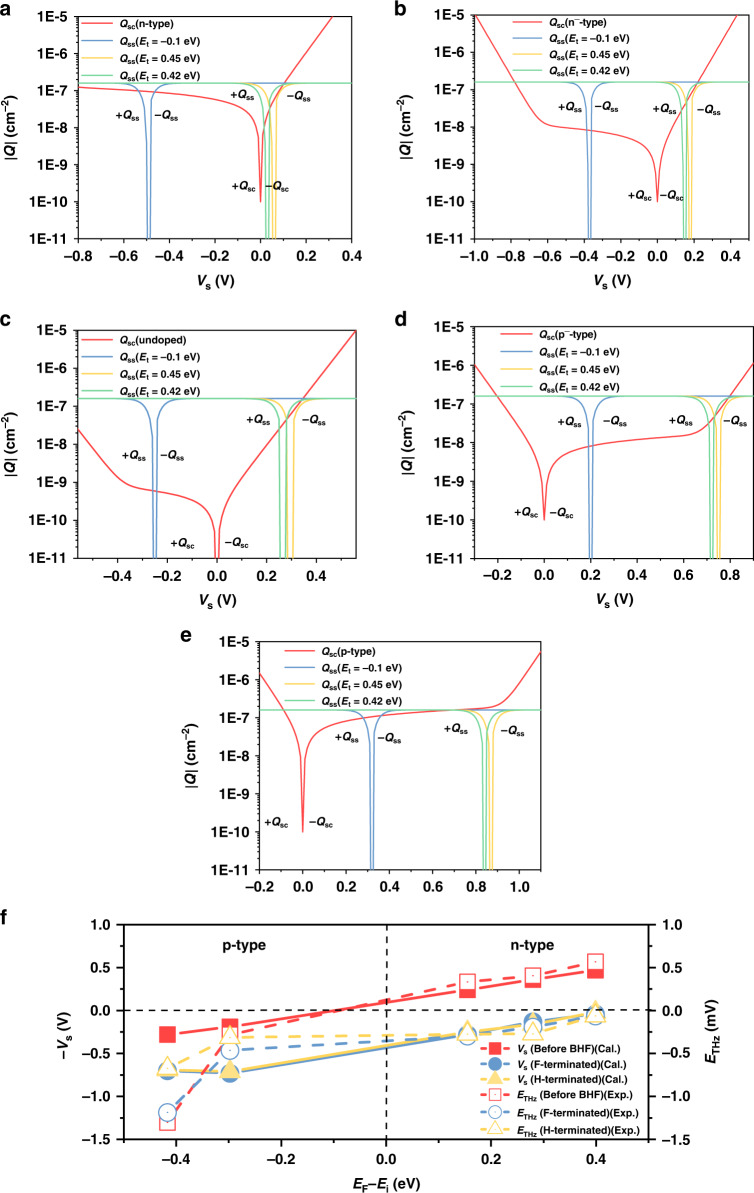
Surface charge calculation and comparison with THz emission. Calculated *Q*_ss_–*V*_s_ and *Q*_sc_–*V*_s_ curves in different surface state energy-level conditions. (*E*_t_ = −0.1, 0.45, and 0.42 eV): (**a**) n-type Si, (**b**) n^−^-type Si, (**c**) undoped Si, (**d**) p^−^-type Si, and (**e**) p-type Si. **f** Comparison between the calculated surface potential and THz emission amplitude for different treatment conditions:before BHF etching, at the F-terminated surface, and at the H-terminated surface

The surface potential under different surface conditions was obtained by plotting both *Q*_ss_ and *Q*_sc_ versus *V*_s_ on the same graph, as shown in Fig. 6a–e, for different doping types and concentrations. Figure 6f shows a comparison between the calculated surface potential and the THz emission amplitude with the change in $E_{{{\mathrm{F}}}} - E_{{{\mathrm{i}}}}$.This figure indicates that the surface potential can be successfully estimated by using the THz emission in the n-type region. In the p-type condition, the polarity of the THz emission reflects downward band bending, but the amplitude variation is inconsistent with the calculated surface potential. This is because of the lower surface state density after BHF etching, which is regarded as a constant in our simulation. Furthermore, the inhomogeneous carrier excitation due to the laser intensity attenuation from the surface to the bulk also has an impact on the THz emission. Since only the photocarriers within the surface electric field are attributed to the THz emission amplitude, the modified THz emission field, including the impact of the penetration depth (*λ*) and thickness of the surface electric field (*w*), is expressed by the following formula:

7 $$\begin{array}{*{20}{c}} {E_{{{{\mathrm{THz}}}}} \propto \mu \frac{{V_{{{\mathrm{s}}}}}}{w}I_{{{\mathrm{p}}}}\left( {1 - e^{ - \frac{w}{\lambda }}} \right)} \end{array}$$

Nevertheless, the penetration depth of 800 nm laser to the Si surface (around 10 μm) is much larger than the thickness of the surface electric field (commonly less than 1 μm), We expect that the carrier excitation is uniform within the surface electric field for simplified estimation. We contend that a semiquantitative estimation of the surface potential can be directly achieved by using THz emission spectroscopy from the Si surface, given its high efficiency and high contrast.

### Application: Mapping the surface potential

**Fig. 7:**
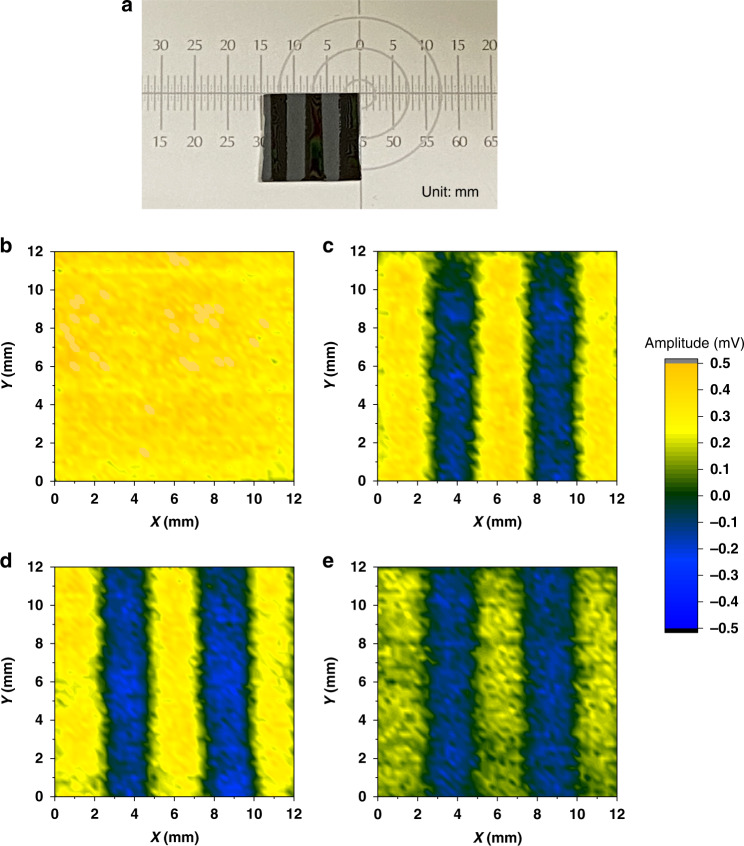
LTEM images of the line-space structure in different conditions. **a** Image of the Si sample with a 2.5 mm interval line-space structure of PR on the surface, which can protect the Si surface from BHF etching. LTEM images of the Si sample (**b**) before etching, after 1% dilute BHF etching for (**c**) 180 s and (**d**) 300 s, and (**e**) after the removal of the PR

In this section, we perform LTEM mapping on the amplitude distribution of THz emission from the Si surface patterned with a line-space structure by photoresist (PR) before and after BHF etching. The size of the line-space structure of the Si sample is shown in Fig. 7a. We used a smaller beam diameter of 1 mm to obtain a higher resolution and a higher laser power of 200 mW to compensate for the decrease in the amplitude resulting from the smaller beam diameter. Figure 7b–d shows the LTEM images before and after 1% dilute BHF etching for 180 and 300 s with PR. An LTEM image after the removal of the PR is shown in Fig.7e. With an increase in the BHF etching time, the native oxide layer was gradually removed, and the H-terminated surface was finally formed. Correspondingly, the polarity of the THz emission amplitude was reversed from positive to negative (turning from yellow to blue in the unprotected area) whereas, in the PR-protected area, the polarity of the amplitude remains positive. After removing the PR, the amplitude of the unetched area became slightly lower owing to the influence of the remaining water at the surface, which can be regarded as a measurement error.

Based on the THz emission amplitude distribution image of the Si surface using LTEM, we can obtain considerable information about the surface potential distribution. First, the surface potential distribution can be quantitatively estimated on a large scale. Because the linear relationship between the THz emission amplitude and surface potential has been verified, a quantitative factor can be experimentally obtained in some special positions and quantitatively estimated for the entire area under the same experimental condition. Second, we can gather semiquantitative information on the surface potential, such as the direction of the band bending and the relative surface potential. The polarity of the THz emission waveform implies that the direction of the surface band bending, and amplitude of the THz emission correspond to the intensity of the surface potential, which means that the surface potential of the sample at different positions can be compared and estimated. In our experiment, the resolution is limited due to the phased-array effect^31^ and the capability of the THz detector. The resolution can be further improved by using a smaller beam spot with a more sensitive near-field detector such as TeraSpike. Furthermore, the LTEM system can also be used to map large-scale images. We have achieved large-scale mapping of the solar cell using a developed LTEM with a higher resolution of 50 μm^50^. Although the solar cell structure is different from the Si surface due to the unique three-dimensional structure with broader depletion layer, leading a higher THz emission amplitude and achieving higher resolution easily, it is an inspiration for the large-scale mapping of Si surface by using LTEM system in the future. These results demonstrate the efficient and semiquantitative application of the LTEM system in rapidly evaluating the surface properties of Si on a wafer scale, providing tremendous value in the development of the modern semiconductor industry.

## Materials and methods

**Fig. 8:**
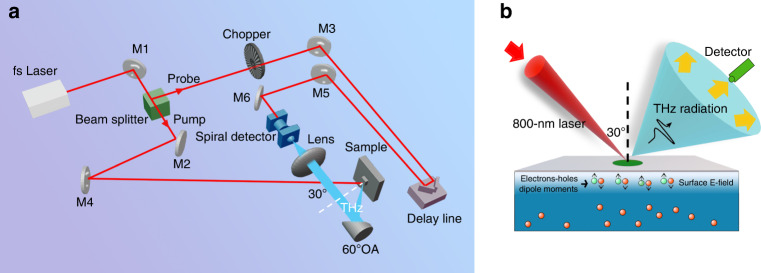
Setup of the TES system and characterization of Si samples. **a** Diagram of the TES system for testing THz emission from the Si samples. **b** Diagram of THz emission characterization from Si samples

**Fig. 9:**
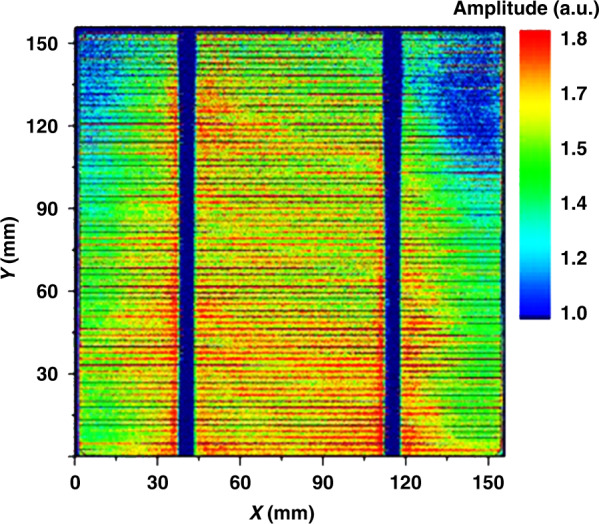
LTEM image of a solar cell. The LTEM image of a large-scale solar cell of 156 mm × 156 mm (781 × 781 pixels reverse bias voltage: 10 V)^50^. (Reproduced from Ref. ^50^, with permission of EU PVSEC)

In this section, the laser-induced TES system and experimental process are introduced. Undoped Si, n-type Si, and p-type Si (100) wafers with different doping concentrations were tested in our experiments. The samples (purchased from Crystal Base co., single-face polished, phosphorus-and boron-doped silicon produced from MIT Co.) and their dopant densities calculated from their resistivity are listed in Table 3. The wafer thickness was ∼525 μm. The sample surfaces were treated with buffered HF solution (HF:NH_4_F = 1:10) for 5 and 60 s, separately, and washed with deionized water to remove the remaining BHF solution on the surface. A dilute BHF solution (1%) was also used to etch the Si surface over a series of etching times. A TES system was used to measure the waveforms and amplitude of the emission from the Si wafer, as shown in Fig. 8a. The samples were excited at 80 MHz using an 800-nm source at an incident angle of 30°^31^, and the THz emission was focused onto the spiral GaAs THz antenna with variable delays. The beam diameter was as large as 5 mm to observe strong THz emission signals from the Si surface. The influence of beam diameters can be found in Fig. S7 in the supplementary information. The pump power was 180 mW and the probe power was 5 mW. The characterization of THz emission from Si samples is shown in Fig. 8b. With the automatic sample stages in the *x* and *y* directions, the TES system can be used as an LTEM system for mapping applications. In the mapping of the line-space structure, we used a smaller beam diameter of 1 mm and an image size of 12 mm × 12 mm (48 × 48 pixels). The large-scale mapping of the solar cell using LTEM with a higher resolution of 50 μm^50^ is shown in Fig. 9. The experimental set-up and details of the solar cell mapping can be found in the supplementary information. These results support the promising ability of LTEM in large-scale mapping and evaluation applications.

**Table 3:** Properties of the Si samples in the experiment

	p^+^–Si	p–Si	p^−^–Si	undoped–Si(n)	n^−^–Si	n–Si	n^+^–Si
Resistivity range (Ω cm)	0.001–0.002	0.1–1	1–10	>1000	1–10	0.1–1	0.001–0.002
Dopant density (cm^−3^)	1 × 10^20^	1 × 10^17^	1 × 10^15^	4 × 10^12^	5 × 10^14^	5 × 10^16^	1 × 10^20^

## Supplementary information

supplementary material
