# Supplementary material for: Rapid, noncontact, sensitive, and semiquantitative characterization of buffered hydrogen-fluoride-treated silicon wafer surfaces by terahertz emission spectroscopy
Source: Light Sci Appl. 2022 Nov 25;11:334. doi: 10.1038/s41377-022-01033-x (PMC9700743; doi:10.1038/s41377-022-01033-x)
Supplement: Supplementary file 1 — supplementary material [file 41377_2022_1033_MOESM1_ESM.docx]

**Supplementary information for:**

**Rapid, noncontact, sensitive and semiquantitative characterization of buffered hydrogen-fluoride-treated silicon wafer surfaces by terahertz emission spectroscopy**

Dongxun Yang^1^, Abdul Mannan^1^, Fumikazu Murakami^1^, Masayoshi Tonouchi^1^*

^1^Institute of Laser Engineering, Osaka University 2-6 Yamadaoka, Suita, Osaka 565-0871, Japan

*Email: [tonouchi.masayoshi.ile@osaka-u.ac.jp](mailto:tonouchi.masayoshi.ile@osaka-u.ac.jp)

**Supplementary Note 1: The laser power dependence on the THz emission amplitude from Si before and after BHF etching.**

Here we provide the laser power dependence on the THz emission before and after 60 s BHF etching. We use an 800-nm fs laser with 80 MHz repetition rate, and the power intensity ranges from 20 mW to 200 mW. We use a beam diameter of 5 mm and the surface power density ranges from 25.5 mW cm^-2^ to 255 mW cm^-2^. The generation rate of the photocarrier per surface area ranges from 1E17 cm^-2^ s^-1^ to 1E18 cm^-2^ s^-1^. The THz emission spectra of different Si samples before and after 60 s BHF etching are shown in Fig.S1 and Fig.S2. The laser power dependences on THz emission amplitude before and after BHF etching are shown in Fig.S3a-b. Here we use the amplitude of second peak to show the THz amplitude to avoid the noise influence on weak signal for better observation. With the laser power increases, the amplitude of THz emission linearly increases and the amplitude ratio between each Si sample with different doping types and doping concentrations are almost the same before and after BHF etching, which reveals the impact of laser power and carrier excitation on the THz emission amplitude and indirectly prove the fact that the surface condition leads to the large variation of the THz emission including amplitude and sign.


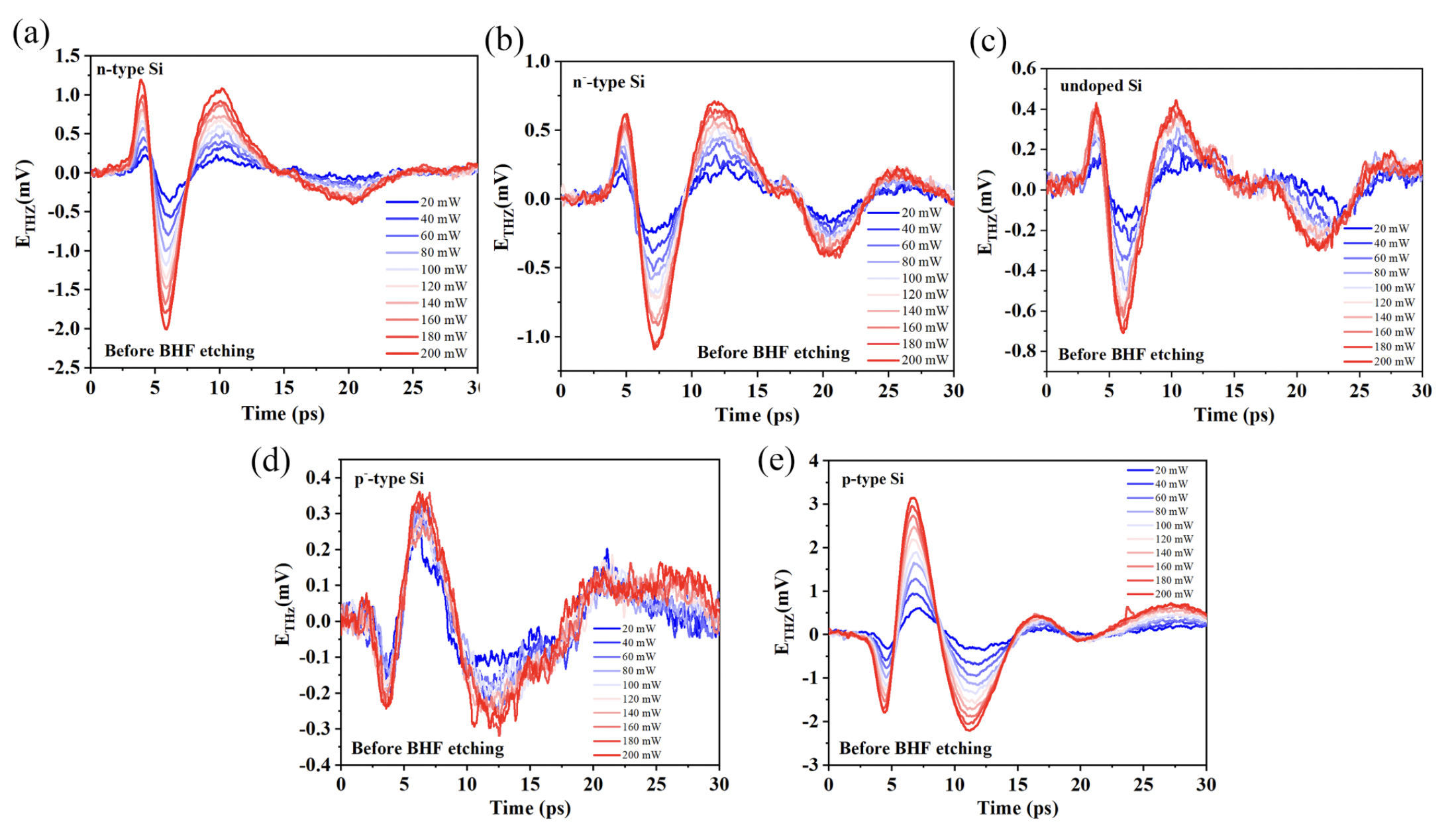


Fig.S1 The THz emission spectra under different laser power from 20 mW to 200 mW from Si samples before BHF etching. (a)n-type Si, (b)n^-^-type Si, (c)undoped Si, (d)p^-^-type Si, (e)p-type Si.


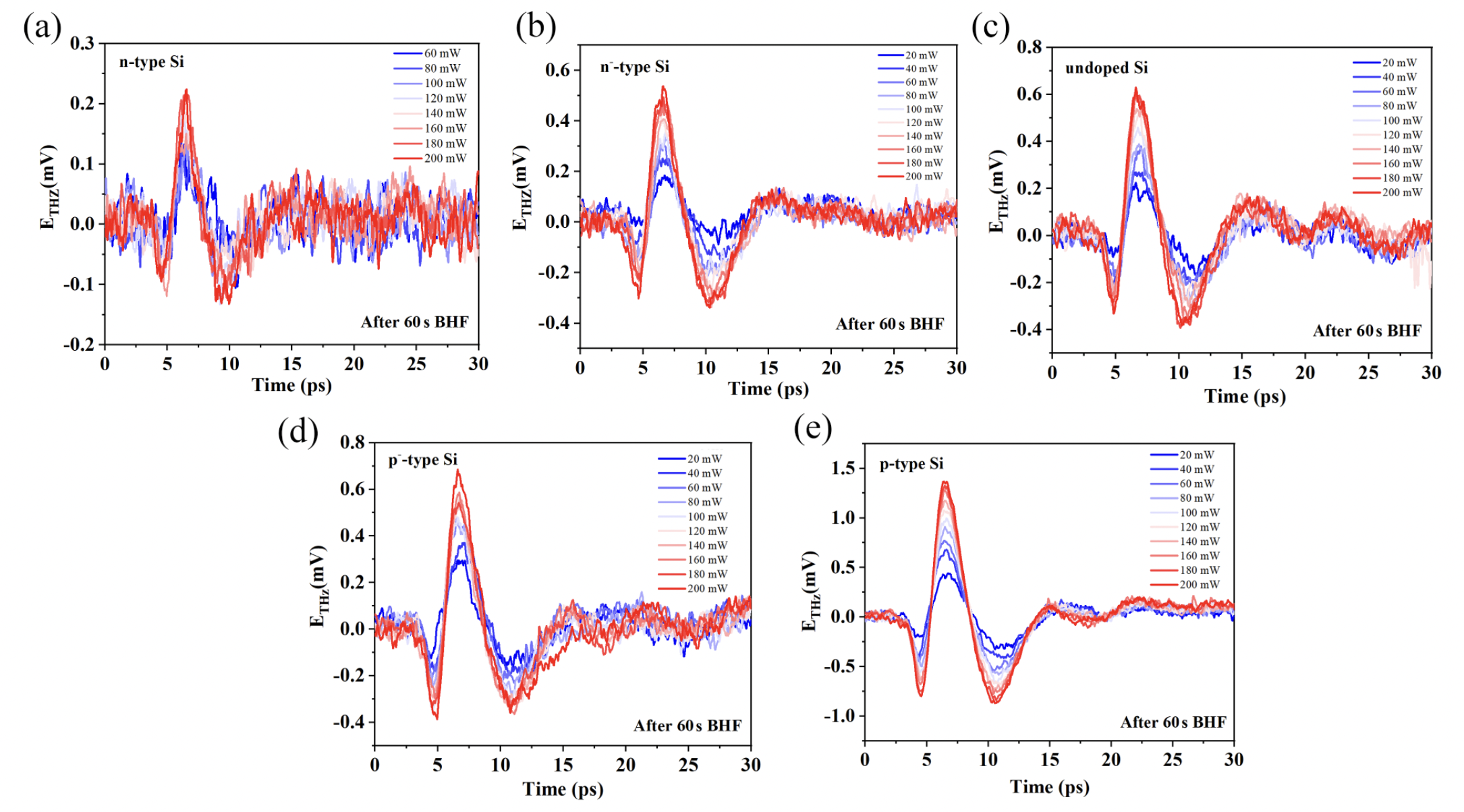


Fig.S2 The THz emission spectra under different laser power from 20 mW to 200 mW from Si samples after 60 s BHF etching. (a)n-type Si, (b)n^-^-type Si, (c)undoped Si, (d)p^-^-type Si, (e)p-type Si.


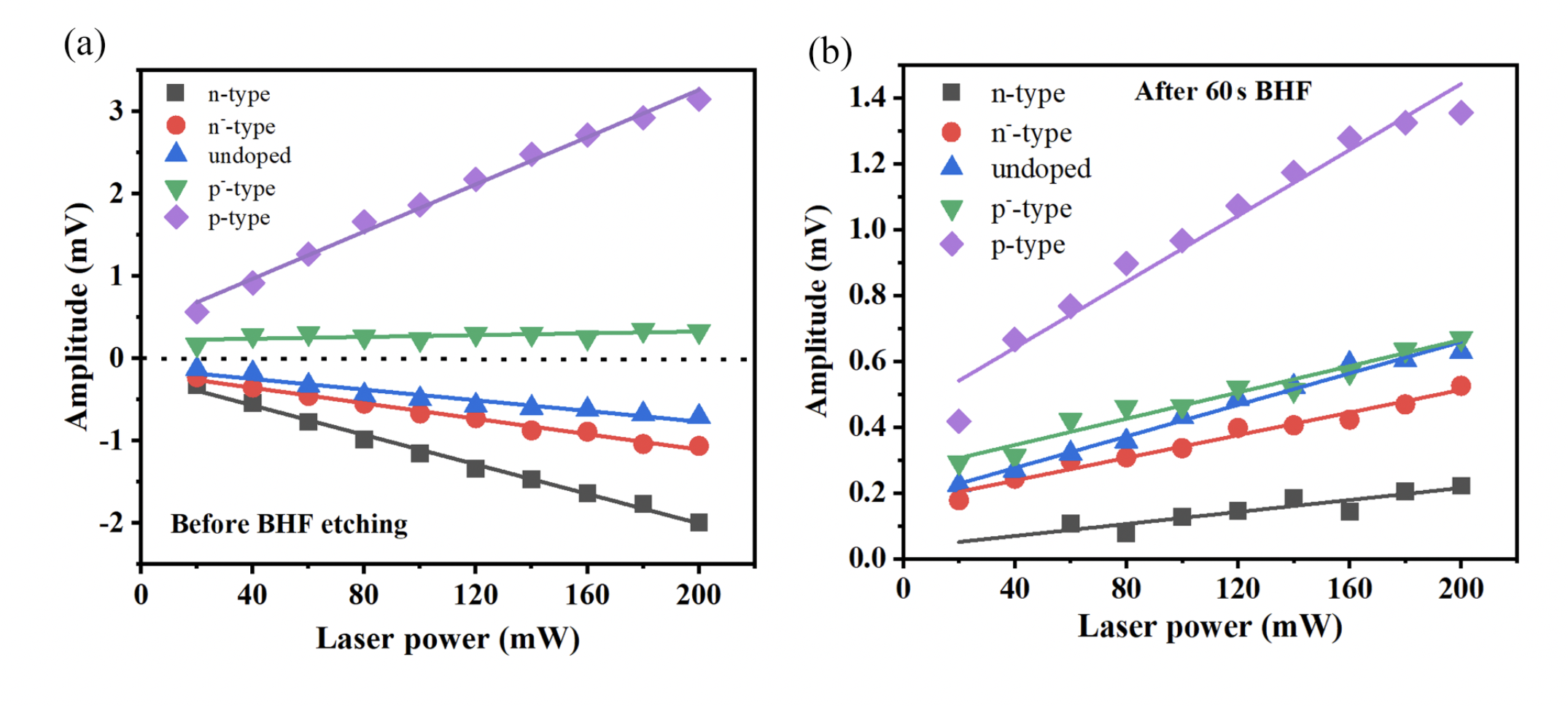


Fig.S3 The laser power dependence on the THz emission amplitude. (a)before BHF etching;(b) after 60 s BHF etching.

**Supplementary Note 2: The impact of laser illumination time on THz emission from H-terminated Si surface.**


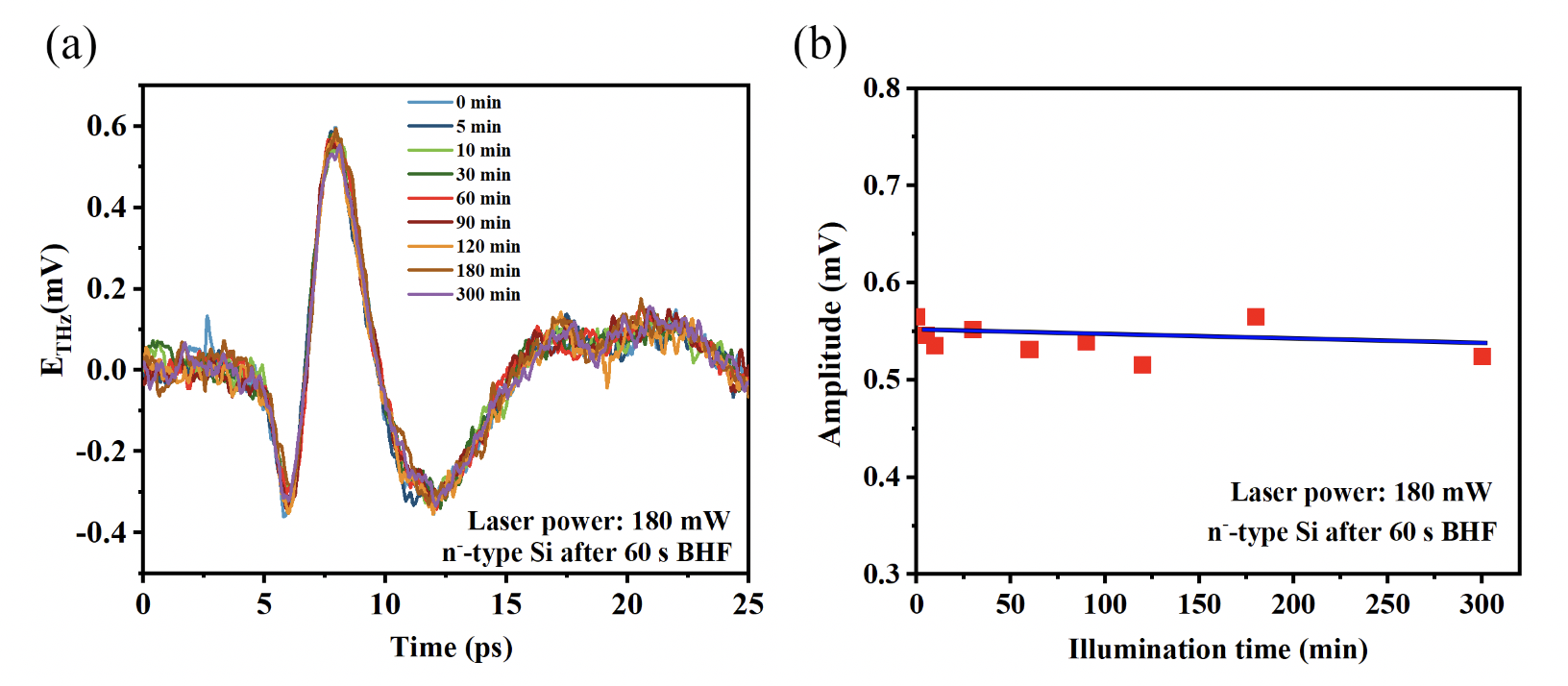


Fig.S4 (a) The THz emission spectra from 60 s BHF etched n^-^-type Si surface after different time of laser illumination from 0 to 300 min. (b) The illumination time dependence on the THz emission amplitude.

Here we provide the THz emission spectra from 60 s BHF etched n^-^-type Si surface after different time of laser illumination from 0 to 300 min and the illumination dependence on the THz emission amplitude in Fig.S4a-b. Based on the experiment results, the amplitude of the THz emission remains almost the same when the laser illumination time increases, which means the H-terminated Si surface is quite stable under laser illumination and the impact of the laser illumination on the Si surface and THz emission is weak. The blue line in Fig.S4b shows the tendency of the amplitude variation with illumination time increases. The decline tendency indicates the long-time dissociation of the hydrogen from the surface with laser illumination^1^.

**Supplementary Note 3: The band diagram of the Si surface**

Here we provide the band diagrams of Si surface with native oxide layer and in F-terminated condition, shows in Fig.S5 and Fig.S6. The native oxide layer provides a negative potential and moves the surface Fermi level below the mid gap, which causes the energy difference between the surface and bulk and leads to the surface band bending. In the F-terminated surface, due to the different electronegative between the fluorine atoms and silicon atoms, the surface dipoles occur and provide additional positive potential, which moves the surface Fermi level close to the conduction band^2^. In this case, the downward bend bending occurs in both n-type and p-type conditions^3^, which leads to the reverse of the THz emission in n-type conditions.


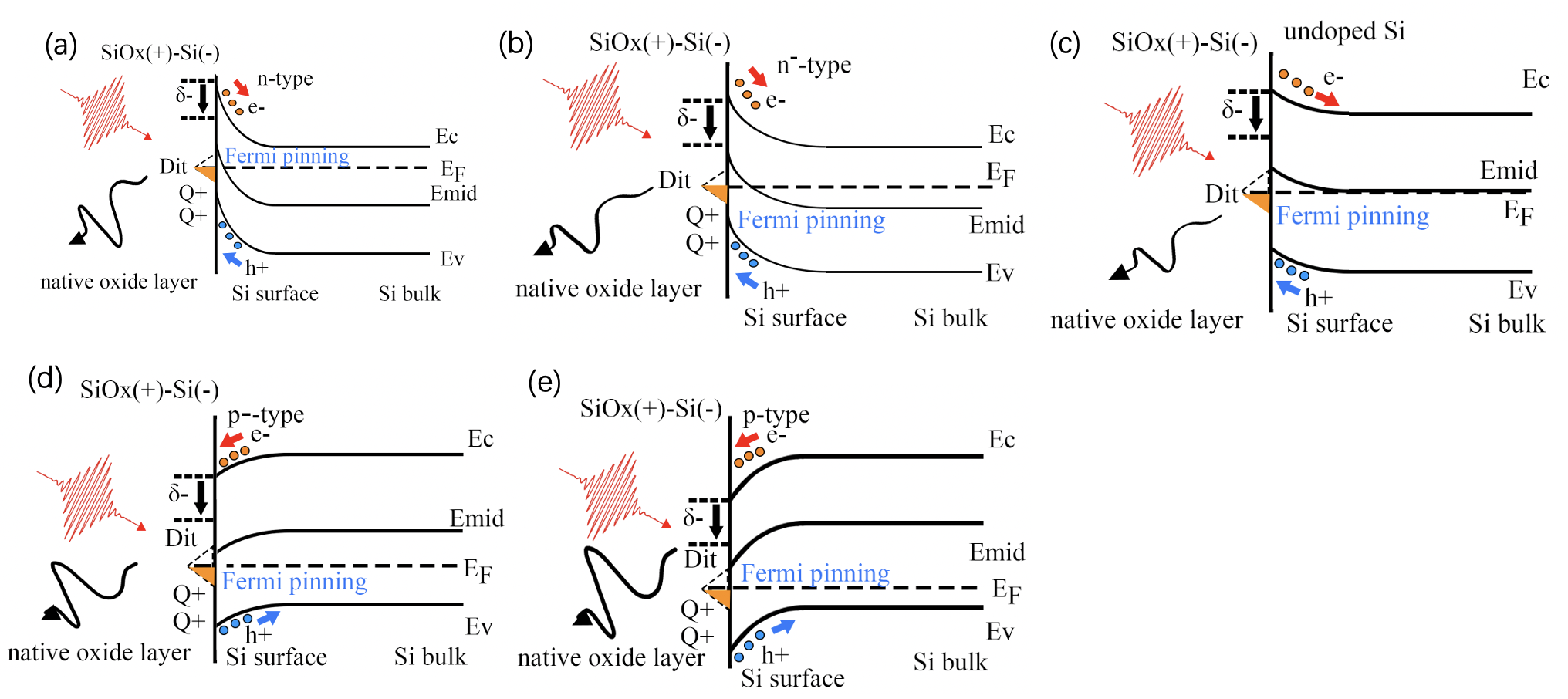


Fig.S5 The band diagram of the Si surface with different doping types and concentrations before BHF etching. (a)n-type Si, (b)n^-^-type Si, (c)undoped Si, (d)p^-^-type Si, (e)p-type Si. Due to the surface dipoles from the H-Si bond at the surface, the surface states energy level changes with $-\delta$ and leads to the surface band bending.


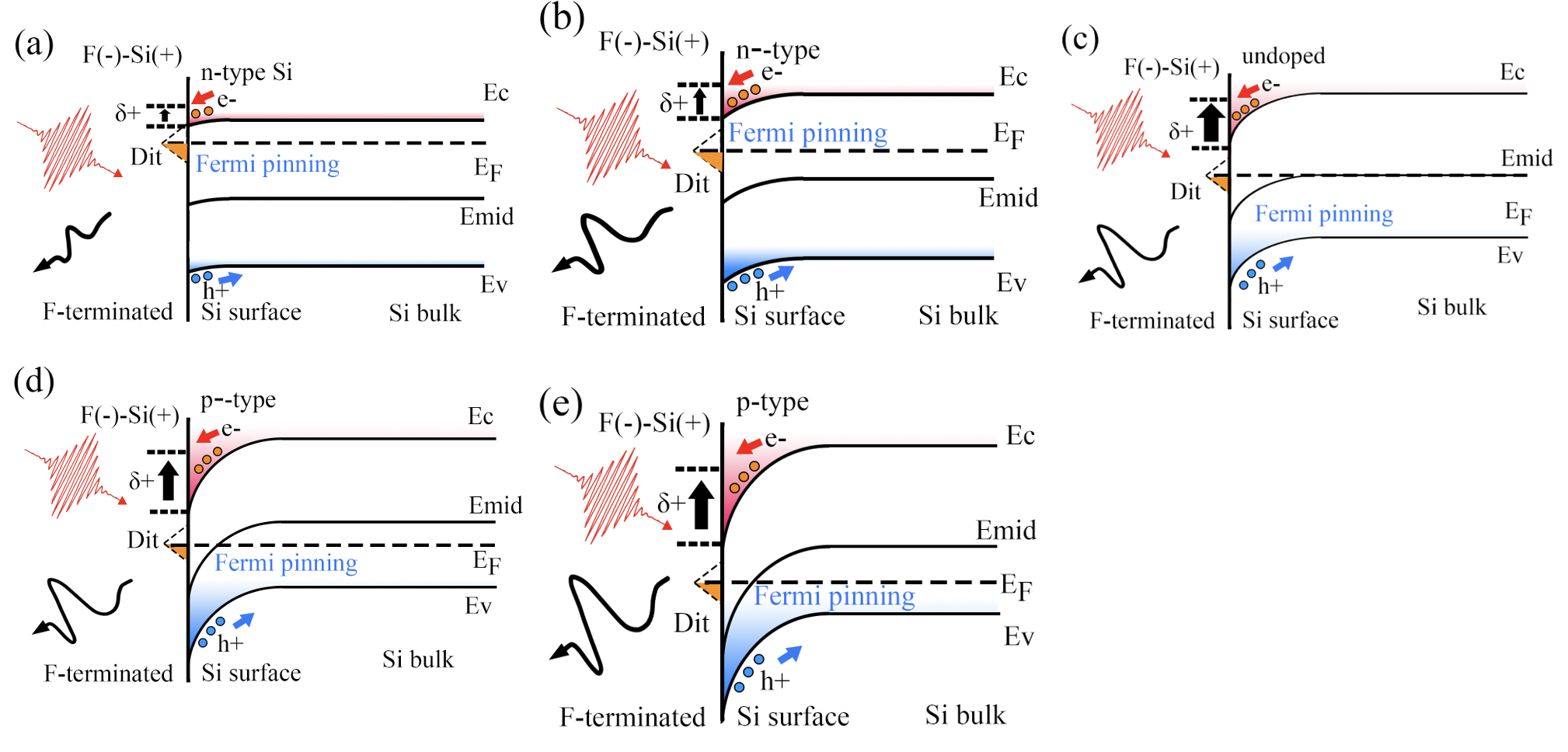


Fig.S6 The band diagram of the F-terminated Si surface with different doping types and concentrations. (a)n-type Si, (b)n^-^-type Si, (c)undoped Si, (d)p^-^-type Si, (e)p-type Si. Due to the surface dipoles from the H-Si bond at the surface, the surface states energy level changes with $+\delta$ and leads to the surface band bending.

**Supplementary Note 4: The THz emission spectra from Si with different beam diameters.**

**
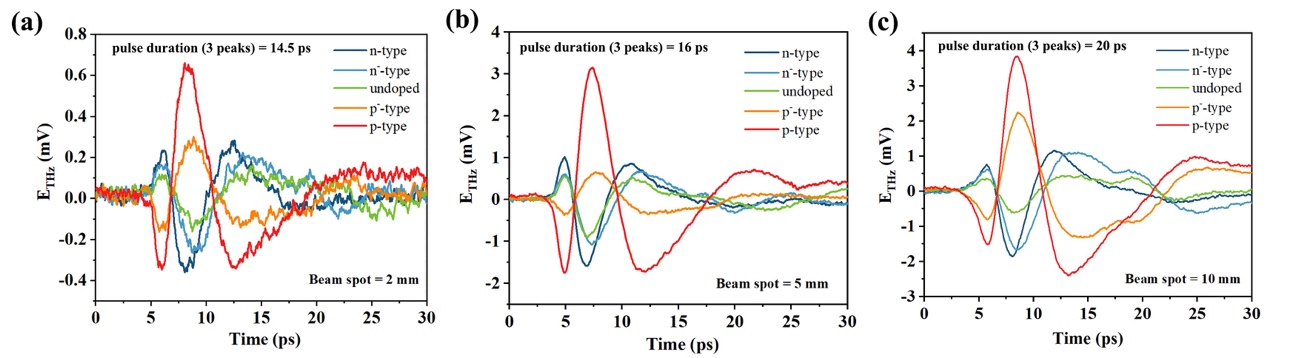
**

Fig.S7 The THz emission spectra from Si samples using different beam diameters of (a)2 mm; (b)5 mm; (c)10 mm.

Here we provide the THz emission spectra from Si before BHF etching with beam diameters of 2 mm, 5 mm and 10 mm, as shown in Fig.S7a-c. Due to the phased-array effect^4^, the intensity of the THz emission amplitude largely decreases with smaller size of beam spot. Meanwhile, the pulse duration is also influenced by the beam spot diameter.

**Supplementary Note 5: Experimental set-up of LTEM mapping on solar cell.**

**
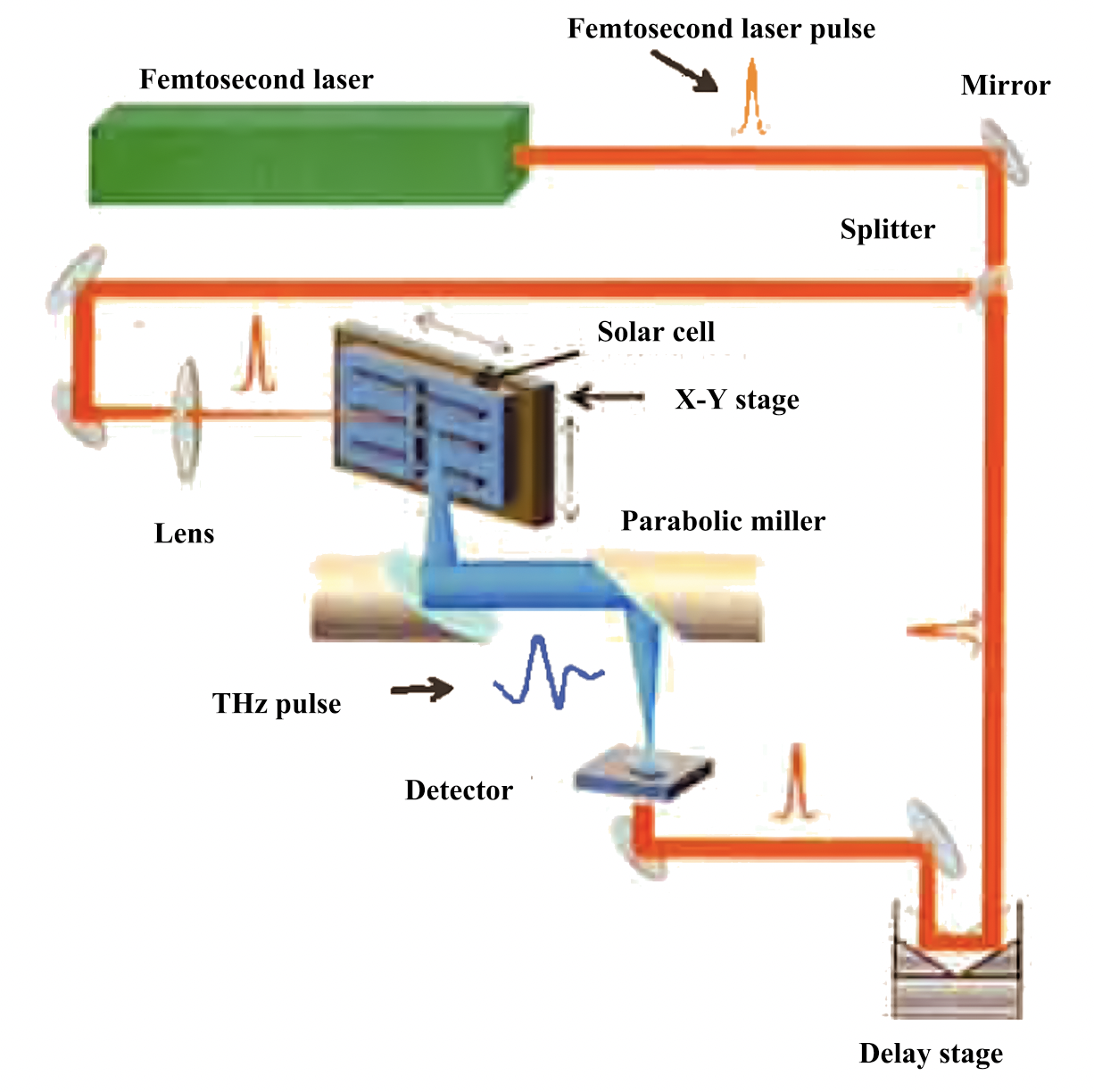
**

Fig.S8 The experimental set-up for mapping solar cell^5^.( Reproduced from Ref. 5, with permission of EU PVSEC)

The large-scale mapping of 156 mm$\times$156 mm solar cells has been successfully achieved by using developed LTEM system^3^, as shown in Fig.S8. We used a mode-locked Ti:sapphire laser (pulse width:100 fs, centre wavelength: 800 nm, repetition rate: 80 MHz) and the laser beam diameter of 50 $\mu$m. The THz waves from the solar cell are radiated into free space and a pair of off-axis parabolic mirrors are set to focus them onto a spiral photoconductive GaAs antenna. The solar cell was measured with a reverse bias voltage of 10 V.

**References:**

1 Murakami, F., Mannan, A., Serita, K., Murakami, H. & Tonouchi, M. Slow optical response of semi-insulating GaN film studied by terahertz emission and photoluminescence spectroscopy. Journal of Applied Physics **131**, 185706, doi:10.1063/5.0086788 (2022).

2 Gleason-Rohrer, D. C., Brunschwig, B. S. & Lewis, N. S. Measurement of the Band Bending and Surface Dipole at Chemically Functionalized Si(111)/Vacuum Interfaces. The Journal of Physical Chemistry C **117**, 18031-18042, doi:10.1021/jp401585s (2013).

3 Watanabe, D., En, A., Nakamura, S., Suhara, M. & Okumura, T. Anomalously large band-bending for HF-treated p-Si surfaces. Applied Surface Science **216**, 24-29, doi:10.1016/S0169-4332(03)00486-0 (2003).

4 Mannan, A. et al. A better understanding of terahertz emission from semiconductor surfaces with a phased-array effect. AIP Advances **11**, 125021, doi:10.1063/5.0077054 (2021).

5 H. Nakanishi, A. I., M. Mizubata, I. Kawayama, H. Murakami, M. Tonouchi. Demonstration of a Novel Inspection System for a Solar Cell Using Terahertz Emission Imaging. Proceedings of 31st European Photovoltaic Solar Energy Conference and Exhibition (EU-PCSEC2015), 511-513, doi:10.4229/EUPVSEC20152015-2DO.4.5 (2015).
